# Effect of Deposition and Protease Digestion on the Ex Vivo Activity of Antimicrobial Peptide-Coated Contact Lenses

**DOI:** 10.3390/nano13020349

**Published:** 2023-01-14

**Authors:** Parthasarathi Kalaiselvan, Debarun Dutta, Nagaraju V. Konda, Savitri Sharma, Naresh Kumar, Fiona Stapleton, Mark D. P. Willcox

**Affiliations:** 1 School of Optometry and Vision Science, UNSW Sydney, Sydney, NSW 2052, Australia; 2 School of Optometry, Aston University, Birmingham B4 7ET, UK; 3 School of Medical Sciences, University of Hyderabad, Hyderabad 500046, Telangana, India; 4 Jhaveri Microbiology Centre, L. V. Prasad Eye Institute, Hyderabad 500034, Telangana, India; 5 School of Chemistry, UNSW Sydney, Sydney, NSW 2052, Australia

**Keywords:** antimicrobial contact lens, deposition, protease digestion, Mel4, cationic peptide, antimicrobial activity, clinical trial

## Abstract

A clinical study of antimicrobial contact lenses containing the cationic peptide Mel4 was conducted. The few adverse events that occurred with this lens occurred on or after 13 nights of wear. The current study examined whether the Mel4 contact lenses lost activity during wear and the mechanism of this loss. Participants wore contact lenses for up to 13 nights. Lenses were tested for their ability to reduce the adhesion of *Pseudomonas aeruginosa* and *Staphylococcus aureus*. The amount of protein and lipid extracted from lenses was measured. The ability of trypsin to affect the antimicrobial activity of Mel4-coated contact lenses was measured. Mel4-coated contact lenses lost their antimicrobial activity at six nights of wear for both bacteria. The amount of lipids (13 ± 11 vs. 21 ± 14 μg/lens at 13 nights wear) and proteins (8 ± 4 vs. 10 ± 3 mg/lens at 13 nights of wear) extracted from lenses was not different between Mel4-coated and uncoated lenses, and was not different after three nights when antimicrobial activity was maintained and thirteen nights when they had lost activity (lipid: 25 ± 17 vs. 13 ± 11, *p* = 0.2; protein: 8 ± 1 vs. 8 ± 4 mg/lens, *p* = 0.4). Trypsin digestion eliminated the antimicrobial activity of Mel4-coated lenses. In summary, Mel4-coated contact lenses lost antibacterial activity at six nights of wear, and the most likely reason was proteolytic digestion of the peptide. Future studies will design and test proteolytically stable peptide mimics as coatings for contact lenses.

## 1. Introduction

Contact lens wear can result in microbially driven adverse events. These can range from frank infection of the cornea, often called microbial keratitis, to ocular surface inflammation associated with microbial colonisation of contact lenses [1]. Collectively, these are called corneal infiltrative events (CIEs). Microbial keratitis has an incidence of 1.2 to 13.0 per 10,000 wearers per year [2]. The incidence of inflammation during contact lens wear, in the absence of frank infection and sometimes called sterile keratitis, is greater at between 1.0 to 44.0 per 100 wearers per year [3]. Microbial keratitis can result in vision loss or even blindness if not appropriately and rapidly treated [3], with the overall rate of vision loss during contact lens wear being 0.56 per 10,000 wearers per year [4,5]. With estimates of at least 140 million lens wearers worldwide [6], these figures equate to about 8000 people a year worldwide losing vision.

Several groups have produced antimicrobial contact lenses that may help to reduce the incidence of microbial and sterile keratitis. The antimicrobial agents in these contact lenses include silver, selenium and other metals, bacterial quorum-sensing inhibitors or cationic peptides [7,8,9]. Contact lenses coated with the cationic antimicrobial peptide Mel4 have progressed to a Phase II/III clinical trial (MACL) and resulted in a reduction in the incidence of CIEs of 69% compared with uncoated control lenses during three months of extended wear (participants wore lenses for 24 h a day for two weeks, then wore new lenses on the same schedule over a three-month period) [10].

Interestingly, all the CIEs that occurred with Mel4-coated lenses occurred on or after 13 nights of wear [10]. This might have been due to a loss of activity of Mel4 peptide at or just before that time point. Prior to wear, the Mel4-coated contact lenses were tested for antimicrobial activity against *P. aeruginosa* and *S. aureus* and showed reduced adhesion to lenses by approximately 2.0 log_10_ colony-forming units (CFU) for both bacteria [10]. A previous study had demonstrated that melimine-coated (the parent peptide of Mel4) [11] antimicrobial contact lenses retained activity following one day (eight hours) of wear, but there was a slight reduction in antimicrobial activity of between 0.5 and 0.8 log10 CFU/lens [12]. Moreover, there was a slight reduction in antimicrobial activity when Mel4-coated silicone hydrogel contact lenses were soaked for 24 h in artificial tear fluid [13]. As the tear film contains components such as proteins, lipids, mucins, electrolytes, salts, peptides and metabolites, these results suggest that the loss of activity may be due to the fouling of the lens surface by one or more of these components or the possibility of degradation of the Mel4 peptide by proteases in tears [14,15,16,17].

The MACL trial used etafilcon A as the base contact lens material [10]. The amount of total protein deposited on the etafilcon A contact lens has been estimated at between 482 μg/lens and 3700 μg/lens [18,19,20,21,22]. The majority of the total protein associated with etafilcon A lens is lysozyme, which is attracted to the etafilcon A lenses due to their negative charge and the positive charge associated with lysozyme [20,23]. Lipid deposition is more commonly seen in silicone hydrogel than hydrogel contact lenses [24,25], but hydrogel contact lenses can bind lipids [19,21,26,27], and etafilcon A lenses adsorb 9.22 to 44.1 μg lipid per lens [19,21,24,25,27].

A previous study had found that melimine (in solution not attached to a lens) retained activity when incubated in tears [28] and this was thought to indicate that this peptide was somewhat protease-resistant and/or did not interact with components in tears. However, tears contain a variety of proteases, such as metalloproteinases (MMP), cathepsins, and cystatins, that may be able to digest Mel4. MMP-8, MMP-9 and cathepsin-S have been found at the highest concentrations in tears [29]. Tryptase from mast cells has trypsin-like activity (i.e., cleaves peptides at arginine (R) or lysine (K) amino acids) and has been found in tears [30]. Interestingly, mast cells, which reside in the conjunctiva, can be stimulated to release their contents by antimicrobial peptides [31]. Neutrophils entering the eye during sleep [32,33] contain several proteases, with one named NSP4 having trypsin-like arginine specificity [34].

This study examined how long the antimicrobial activity of Mel4-coated contact lenses was retained during wear, quantified the amount of protein and lipid deposited on the worn Mel4-coated contact lenses, and assessed the ability of trypsin to reduce the antimicrobial activity of Mel4-coated contact lenses.

## 2. Materials and Methods

### 2.1. Mel4 Antimicrobial Contact Lenses and Its Activity

Etafilcon A contact lenses (Acuvue 2; Johnson & Johnson Vision Care, Jacksonville, FL, USA) were used for this study. Mel4 peptide (amino acid sequence KNKRKRRRRRRGGRRRR; American Peptide Company, Sunnyvale, CA, USA) was synthesized by conventional solid-phase peptide synthesis with >95% purity. The procedure for covalently attaching Mel4 to contact lenses by creating an amide bond between the amine groups of Mel4 and the carboxylic acid group of the contact lens, and preparing control lenses, has been reported in previous studies [10,35]. Before dispensing the study lenses to the participants, the antimicrobial activity of coated lenses was assessed by bacterial adhesion assay [10,36].

### 2.2. Study Participants

The study was approved by the Human Research Ethics Committee of the University of New South Wales (HC17706) and the Institutional Ethics Committee of Hyderabad Eye Research Foundation (LEC 07-17-066) at L.V. Prasad Eye Institute. In addition, the clinical trial was registered with the Clinical Trials Registry—India (CTRI Trial ID CTRI/2017/09/015296). The study procedures were conducted according to the tenets of the Declaration of Helsinki. The inclusion and exclusion criteria were identical to a previous study [10]. Twelve participants were recruited (the minimum number needed to show significant differences in bacterial adhesion for a decrease of 1.5 log_10_ in bacterial adhesion on Mel4-coated lens compared to control lenses at the 5% level of significance and 80% power after adjusting for a 20% dropout rate).

### 2.3. Study Design

The study design was a prospective, randomized, contralateral design to evaluate the ex vivo antiadhesion activity of Mel4 peptide-attached commercially available hydrogel contact lenses against ocular bacteria. Participants were randomized (using a computer-generated random allocation table) to wear Mel4-coated antimicrobial contact lenses in one eye and an uncoated etafilcon A control lenses in the contralateral eye.

### 2.4. Clinical Procedures

Twelve participants who had completed the MACL clinical trial study [10] successfully without any adverse events were recruited. A baseline visit was conducted to examine their ocular health, ocular refraction and suitability for enrolment in the study, and informed consent was obtained from those who were suitable. A total of six sets of contact lenses were allocated to each subject at the baseline visit. To reduce the possibility of participants mixing right and left eye lenses, all right eye and left eye lens vials were affixed with green and white labels, respectively, with their study ID numbers. Each participant wore a new set of contact lenses as per the study protocol during their study visits. If the participants needed to remove their lenses temporarily, they were given Biotrue contact lens care solution (Bausch and Lomb, Rochester, NY, USA) and a lens case only for temporary storage.

Following the baseline visit, the study required up to four visits: after eight hours of lens wear (visit 1), one night of extended lens wear (visit 2), three nights of extended lens wear (visit 3) and six nights of extended lens wear (visit 4). Contact lenses were collected aseptically at each visit (the rest of the day, the subjects were instructed not to wear contact lenses but to wear spectacles for vision correction). The next day, subjects wore a new set of contact lenses for the required time period. At each follow-up visit, worn lenses were collected aseptically and added to a glass vial containing 2 mL sterile phosphate buffered saline (PBS) at pH 7.4 and stored in −80 °C. Ten subjects were asked to repeat wearing lenses for three nights of extended wear so that these lenses could be assessed for the amount of protein and lipid deposited. In addition, 20 study lenses of 10 participants in the MACL clinical trial [10] were collected aseptically following 13 nights of extended wear. Three unworn Mel4-coated and control lenses were taken as control lenses.

### 2.5. Retention of Antimicrobial Activity against Bacterial Strains

The unworn Mel4-coated and worn control lenses (uncoated) and Mel4-coated contact lenses were processed less than 48 h after lens collection for the evaluation of retention of antimicrobial activity. The retention of antimicrobial activity after lens wear was calculated based on a reduction in the adhesion of viable bacteria (*P. aeruginosa* ATCC 27853 (originally isolated from blood) and *S. aureus* L2260/15 (originally isolated from the cornea of a patient with keratitis) to worn Mel4-coated contact lenses compared to the worn control lenses. The bacterial adhesion protocol has been reported in prior studies [10,36,37]. Briefly, bacteria were grown overnight in trypticase soya broth (Becton Dickinson and Company, Sydney, Australia), then washed and resuspended to 1.0 × 10^6^ CFU mL^−1^ in phosphate-buffered saline (PBS; pH 7.4) for *P. aeruginosa* ATCC 27853 or 1/10 diluted trypticase soya broth for *S. aureus* L2260/15 and 1 mL of each was transferred to the wells of 24-well tissue culture plates containing Mel4-coated or control lenses. The plates were incubated for 18 h with shaking (120 rpm) at 37 °C. The contact lenses were then washed three times with 1× PBS and then stirred in a vortex mixer rapidly in 2 mL of fresh 1× PBS containing a small magnetic stirring bar for a minute. The resulting lens slurry was serially diluted (1/10), and 3 × 20 μL of each dilution was transferred to nutrient agar for the recovery of cells. After incubation for 18 h at 37 °C, viable microorganisms were enumerated as CFU mm^−2^ of a lens.

### 2.6. Extraction and Analysis of Protein Deposits from Contact Lenses

Protein extraction has been reported previously [38]. Briefly, 1.5 mL of protein extraction solution (50:50 mix of 0.2% trifluoroacetic acid and acetonitrile (ACN/TFA)) was added to the glass vials containing contact lenses and incubated in darkness at room temperature for 24 h at 50 rpm in a shaker incubator. The extracts were removed from the glass vials and transferred to sterile Eppendorf tubes and lyophilized to dryness overnight in a Savant Speed-Vac (Thermo Scientific, San Jose, CA, USA). Dried protein pellets were stored at −80 °C prior to the quantification of proteins. To quantify the total amount of protein in the deposits [39], the dried proteins from contact lenses were diluted in 1 mL PBS and an aliquot was mixed with the bicinchoninic acid working reagent as per the manufacturers’ instructions (Pierce™ BCA, ThermoFisher Scientific, North Ryde, NSW, Australia). After incubation at 37 °C for 30 min, the absorbance of the solution was measured at 562 nm using a spectrophotometer (FLUOstar Omega, BMG Labtech, Offenburg, Germany) and compared to a standard curve of dilutions of bovine serum albumin.

Aliquots of the protein extracts in PBS were subjected to sodium dodecyl sulfate-polyacrylamide gel electrophoresis (SDS-PAGE) using a NuPAGE^®^ electrophoresis system (Thermo Scientific, Waltham, MA, USA). Proteins were separated in precast NuPAGE^TM^ 4–12% bis-Tris Midi Protein gels using NuPAGE (3-(N-morpholino)propanesulfonic acid SDS running buffer (NuPAGE MPOS SDS; Thermo Scientific). Prestained SDS-PAGE broad-range standards (Bio-Rad Laboratories Inc., Hercules, CA, USA) were used as molecular weight markers. All samples were diluted into NuPAGE LDS sample buffer and NuPAGE sample reducing agent. The mixture was heated to 70 °C and the whole volume was immediately applied onto the gel. Electrophoresis was run in MOPS running buffer, at a constant voltage of 200 V for 55 min. After electrophoresis, gels were stained with colloidal Coomassie blue R-250 (Bio-Rad Laboratories Inc.). Stained proteins on the gel were scanned with MagicScan software on Imagescanner (Amersham Pharmacia Biotech, Piscataway, NJ, USA).

### 2.7. Extraction and Analysis of Lipid Deposits from Contact Lenses

Thirty-two study contact lenses of 16 subjects who had worn lenses for two weeks of extended wear in the MACL study [10] and 10 contact lenses of five subjects following three nights of extended wear were collected aseptically. Lenses were placed in individual sterile glass vials and stored at −80 °C. Three unworn Mel4-coated and control lenses were used as controls. The lipid extraction procedure has been reported previously [40]. Briefly, 2 mL extraction solution consisting of tertbutyl methyl ether (MTBE):methanol (10:3 vol/vol; containing 0.01% butylated hydroxytoluene) was added to the lenses in glass vials and the vials were shaken at 50 rpm for two hours. Aliquots were removed carefully without breaking the contact lens and placed in a sterile glass vial. Biphasic lipid extraction was performed by adding 500 μL of 0.15 M ammonium acetate to the MTBE:methanol solution and the glass vials were vortexed and spun at 3500 g for 15 min to allow complete phase separation. The upper organic layer was removed and added to a new glass vial, dried under a stream of nitrogen gas at room temperature and stored at −80 °C freezer prior to quantification of lipids.

Individual lipids for a standard mixture were purchased from Avanti Polar Lipids Inc. (Alabaster, AL, USA) and Nu-Chek Prep Inc. (Elysian, MN, USA). An internal standard lipid solution was made based on the current understanding of the major lipid classes present in human tears [41] and total lipids extracted from etafilcon A lenses [21]. The sulfo-phospho-vanillin assay was adapted with modification to quantify total lipids in lens extracts [42]. Briefly, the dried lipid samples and dilutions of the standard lipid solution were incubated with 120 μL of 95% sulfuric acid at 95 °C for 20 min in individual glass vials. The reaction mixture was rapidly cooled by placing the vials on the crushed ice for five minutes. The contents of each glass vial were vortexed for one minute and 100 μL was transferred to individual wells of a 96-well plate. After the addition of 50 μL of sulfo-phosphoric-vanillin acid agent containing 0.2 mg per mL vanillin (in 17% aqueous phosphoric acid), the 96-well plate was incubated at room temperature for 10 min in the dark. After incubation, the absorbance of the solution was measured at 535 nm using a spectrophotometer. A standard curve was established from the dilutions of the internal standard lipid mixture and used to quantify the lipids extracted from the lenses.

### 2.8. The Effect of Trypsin on the Antimicrobial Activity of Mel4

Using the Expasy peptide cutter module (https://web.expasy.org/peptide_cutter/, accessed on 20 November 2022), Mel4 (KNKRKRRRRRRGGRRRR) was predicted to be susceptible to cleavage by arg-C proteinase, clostripain, lysC, lysN and trypsin. Therefore, sequencing grade trypsin (Promega Corp, Madison, WI, USA) was dissolved in 50 mM acetic acid (trypsin buffer, pH 3.0; Promega Corp.) to 0.5 µg µL^−1^. Mel4 (8 mg) was added to 160 µL of the trypsin suspension and this mixture was then added to 1340 µL of Muller–Hinton broth (MHB; Oxoid) containing 0.2% *w*/*v* bovine serum albumin (Sigma Aldrich, St. Louis, MI, USA) and 0.01% *v*/*v* acetic acid (Sigma Aldrich) to obtain a final volume of 1.5 mL. This mixture was incubated for 18 h at 37 °C in an orbital shaker (80 rpm) before performing the antimicrobial activity analysis. Controls used the same conditions but in the absence of trypsin.

Using a previously reported protocol with modification [43], the minimum inhibitory concentration (MIC) of trypsin-digested and undigested Mel4 was determined against *P. aeruginosa* ATCC 27853 and *S. aureus* L2260/15. Briefly, bacterial strains were grown in MHB for 18 h at 37 °C. Following incubation, bacteria were washed three times in PBS and diluted into fresh MHB containing 0.2% *w*/*v* bovine serum albumin and 0.01% *v*/*v* acetic acid. The turbidity of the bacterial suspensions was adjusted to OD_660 nm_ 0.1 (which gave 1 × 10^8^ colony-forming units (CFU) mL^−1^ on retrospective agar plate counts) and then further diluted to achieve 5 × 10^5^ CFU mL^−1^ as a final bacterial concentration. Trypsin-treated Mel4 and untreated Mel4 were serially diluted from 4000–7.8 μg mL^−1^ and 100 μL of bacterial inoculum containing 5 × 10^5^ CFU mL^−1^ bacteria were added in each well of 24 tissue culture well plates. Wells containing bacteria with no peptide or no bacteria or peptide acted as controls. The suspensions were incubated at 37 °C with shaking at 120 rpm for 18 h. After incubation, the absorbance of each well was measured at 660 nm by a spectrophotometer. The well at the lowest concentration without any bacterial growth was considered to be a minimum inhibitory concentration.

As the Mel4-coated contact lenses had been previously shown to contain 62.6 μg of Mel4 per lens [10], they (n = 3) were added to 300 mL of PBS and 15 µL of 0.5 µg µL^−1^ trypsin was then added to each sample. All the samples were incubated at 37 °C for 18 h in a shaking rotator (50 rpm). After incubation, the lenses were washed three times with PBS. Each lens was used to test the activity against *S. aureus* using the previously described bacterial adhesion assay [10,36,37]. Uncoated and Mel4-coated lenses were used as negative and positive controls, respectively.

### 2.9. Statistical Analysis

Data were analysed using Microsoft^®^ Office Excel^®^, and Graph Pad Prism 7.02 (Graph Pad Software Inc., San Diego, CA, USA). The normality of the data was checked using the Shapiro–Wilk test. Normally distributed variables are described using means and standard deviation and abnormally distributed variables were described using medians with ranges. The bacterial adhesion to the contact lens data was log_10_ (x + 1)-transformed prior to analysis and x denotes the number of bacteria adherent to the contact lenses in CFU mm^−2^. A comparison of total protein and lipid deposits between Mel4-coated and control lenses was analysed with paired *t*-test or Wilcoxon signed-rank test. A comparison between lenses worn for three vs. 13 nights, trypsin-treated and untreated Mel4-coated contact lenses were analysed with the Mann–Whitney U test. For all tests, the level of statistical significance was maintained at *p* < 0.05.

## 3. Results

### 3.1. Retention of Antimicrobial Activity

**Figure 1:**
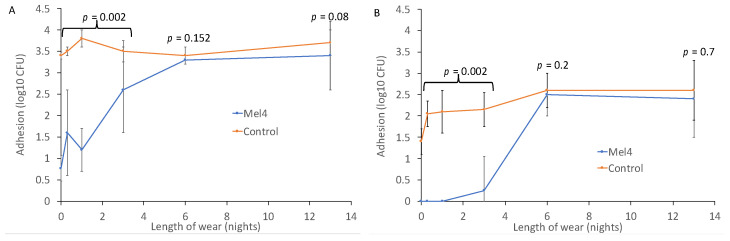
Bacterial adhesion to Mel4-coated and control contact lenses worn for up to 13 nights for (**A**) *P. aeruginosa* and (**B**) *S. aureus*.

Prior to wear, Mel4-coated contact lenses reduced the adhesion of *P. aeruginosa* by 2.63 log_10_ CFU, and of *S. aureus* by 1.4 log_10_ CFU (Figure 1). The reduction in the adhesion of *P. aeruginosa* remained significant up to and including three nights of lens wear, with an average reduction of 1.8 log_10_ CFU (*p* = 0.002). Similarly for *S. aureus*, the reduction in adhesion prior to lens wear was 1.4 log_10_ CFU, and this was maintained with an average reduction of 2.0 log_10_ CFU up to three nights of wear (*p* = 0.002). For both *P. aeruginosa* and *S. aureus*, wearing lenses for six or 13 nights eliminated the antimicrobial effect of Mel4 (Figure 1).

Interestingly, for *S. aureus* there was a gradual increase in adhesion to the control contact lenses. Unworn control lenses had 1.4 ± 0.3 log_10_ CFU of *S. aureus* bound to them, and this increased to 2.0 ± 0.3 log_10_ CFU after eight hours of wear during the day, 2.1 ± 0.5 log_10_ CFU after one night of wear, 2.2 ± 0.4 log_10_ CFU after three nights of wear and 2.6 log_10_ CFU after six and 13 nights of wear. This increase in adhesion might have been due to the deposition of proteins or lipids onto the lenses during wear.

### 3.2. Deposition on Contact Lenses

**Figure 2:**
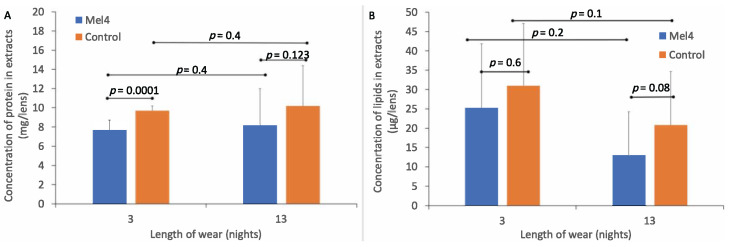
Amount of protein (**A**) and lipid (**B**) deposited on Mel4-coated and control lenses during wear.

The standard curve of absorbance vs. concentration of BSA had a best fit line of R² = 0.9829 and formula for converting absorbance into protein (BSA) concentration of y = 0.0014x + 0.1276. The amount of total protein extracted from Mel4-coated and control contact lenses is shown in Figure 2A. The amount of protein extracted from control lenses (9.7 ± 0.5 mg/lens) was greater than from Mel4-coated lenses (7.7 ± 1.0 mg/lens) after three nights of lens wear, and this difference was significant (*p* < 0.0001). Similarly, after 13 nights of lens wear, the amount of total protein in lens extracts was greater for control lenses (10.2 ± 4.2 mg/lens) compared to Mel4-coated lenses (8.2 ± 3.8 mg/lens), but this was not significantly different (*p* = 0.123). The difference in the amount of protein deposited after three or 13 nights of wear was not significant (*p* = 0.4).

**Figure 3:**
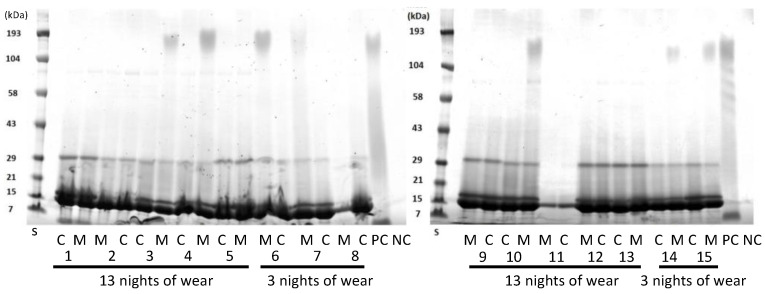
SDS-PAGE of extracts from worn etafilcon A contact lenses. C = control lenses; M = Mel4-coated lenses; numbers on the x-axis denote different patients. S = standards of known molecular mass.

SDS-PAGE was used to separate the proteins in the lens extracts as a way of evaluating whether the proteins extracted were similar or different between the lens types (Figure 3). As the lenses were paired, that is, a Mel4 lens was worn by the same person in one eye and a control lens in the other eye, this allowed for direct comparison for individual participants. Generally, all lens extracts contained a major band at 14 kDa, the next most intense band at 18 kDa, and then a band at 30 kDa. Some lens extracts contained a band at 80 kDa and a diffuse band at 190 kDa. There did not appear to any difference between the protein bands extracted from control or Mel4-coated contact lenses. Usually, the control and Mel4-coated lens extracts looked identical, most easily seen for participant 11 (Figure 3). The lens extract from Mel4 lenses (positive control) also had the diffuse band at 190 kDa, as well as diffuse bands or a smear between 100 and 21 kDa. No bands were seen from the extract of uncoated etafilcon A lenses.

### 3.3. Total Lipid Deposition on Lenses

The standard curve for absorption vs. lipid concentration had a best fit line of R² = 0.9902 and the formula for estimating the amount of lipid in lens extracts was y = 0.0035x–0.001. The amount of total lipids extracted from Mel4-coated and control contact lenses is shown in Figure 2B. There was a lower concentration of total lipids extracted from Mel4-coated lenses (25.23 ± 16.62 μg/lens) compared to control lenses (30.99 ± 16.12 μg/lens) after three nights of lens wear, but this difference was not significant (*p* = 0.6). Similarly, after 13 nights of lens wear, there was a lower concentration of total lipids extracted from Mel4-coated lenses (13.11 ± 11.08 μg/lens) compared to control lenses (20.82 ± 13.82 μg/lens) and again this difference was not significant (*p* = 0.08). There was a higher concentration of lipids extracted from both lens types after three compared to 13 nights of lens wear but there were no differences in the concentration of lipids deposited between three and 13 nights of lens wear for Mel4-coated (*p* = 0.1) or control lenses (*p* = 0.2).

### 3.4. Effect of Trypsin on Mel4

Untreated Mel4 had MIC of 62.5 μg mL^−1^ against *P. aeruginosa* and 125 μg mL^−1^ against *S. aureus*. However, the MIC for trypsin-digested Mel4 increased by at least 64-fold against *P. aeruginosa* and 32-fold with *S. aureus,* as at the highest concentration of Mel4 used (4000 μg mL^−1^) trypsin-digested Mel4 did not inhibit the growth of either bacterium. This suggests that Mel4 had lost all its antimicrobial activity when treated with trypsin.

**Figure 4:**
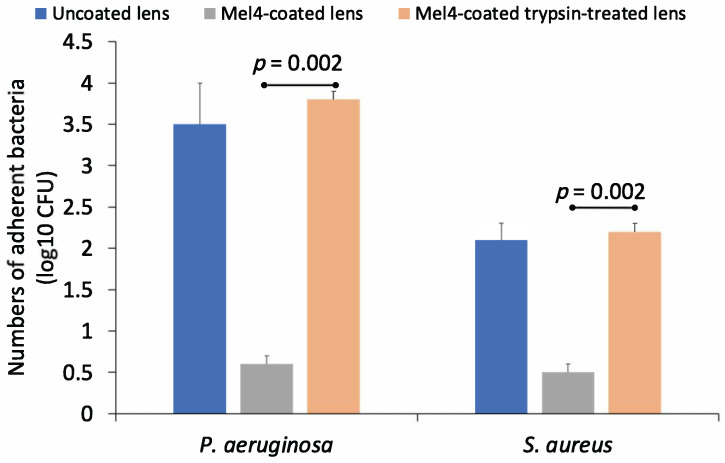
Effect of trypsin treatment on the numbers of *S. aureus* cells adhering to contact lenses coated with Mel4.

The antimicrobial activity of trypsin-treated and untreated Mel4-coated contact lenses against *P. aeruginosa* and *S. aureus* is shown in Figure 4. Mel4-coated contact lenses that had not been treated with trypsin retained antimicrobial activity against both bacteria. However, trypsin treatment resulted in a loss of activity and an 83% reduction in adhesion of *P. aeruginosa* and a 76% reduction in the adhesion of *S. aureus* to trypsin-treated Mel4-coated contact lenses compared to untreated Mel4-coated contact lenses. Trypsin-untreated Mel4-coated lenses showed a significantly (*p* = 0.002) lower amount of bacterial adhesion compared to trypsin-treated Mel4-coated lenses.

## 4. Discussion

Analysis of data from a Phase II/II clinical trial of Mel4-coated contact lenses had shown that all the corneal infiltrative events that occurred with Mel4-coated lenses occurred on or after 13 nights of wear [10]. This prompted the current evaluation of whether Mel4-coated lenses had lost their ability to prevent bacterial adhesion sometime during the wear period, and if so, the mechanisms that might have contributed to this.

The current study provides the first evidence that Mel4-coated contact lenses retained antimicrobial activity after at least three nights of extended wear but lost activity at six nights of extended wear. In addition, the current study found that a likely explanation for the loss of antimicrobial activity of Mel4-coated lenses was protease digestion of the Mel4 peptide.

Previous studies had shown some slight reductions in the activity of melimine-coated lenses after one day of wear [12] or for Mel4-coated lenses after 24 h of soaking in artificial tear fluid [13]. Apart from studies examining melimine or Mel4-containing contact lenses in human clinical trials [9,11,12], there is only one other peer-reviewed paper that has reported on human wear of an antimicrobial contact lens [44], but that study did not report on retention of activity after wear and also had not used a cationic peptide. After eight hours of lens wear, Mel4-coated contact lenses retained approximately 2.0 log_10_ CFU inhibition of adhesion of *P. aeruginosa* and *S. aureus*. This is in agreement with a previous study [12] where melimine-coated contact lenses retained 1.5 log_10_ inhibition against *P. aeruginosa* and *S. aureus* after eight hours of contact lens wear, even though different bacterial strains of each species were used for the assays.

Interestingly, in the current study, there was an increase in adhesion of *S. aureus* to the control lenses during the course of lens wear (Figure 3). Lens wear can increase the adhesion of *S. aureus*, although this may be lens- and strain-dependent [45]. The increase in adhesion may be due to the adsorption of lysozyme, which can increase the adhesion of *S. aureus* to etafilcon A lenses in vitro [46].

The gradual decrease in the inhibition of bacterial adhesion as the number of wearing nights increased was hypothesized to be due to the deposition of human tear film components such as lipocalin, lactoferrin, albumin, lysozyme, cholesterol and phospholipid to lenses [46,47,48,49,50,51]. These might either mask the Mel4-coating or interact directly with Mel4 due to their charge. For example, lipocalin is the predominant negatively charged protein in tears and so has the potential to interact with the covalently attached positively charged Mel4. Previous ex vivo studies have reported that the total protein deposited on etafilcon A contact lenses after 14 days of daily wear ranged between 0.5 mg/lens and 3.7 mg/lens [21,26,52]. Only one study followed the same method of the current study to extract and quantify the proteins extracted from the worn contact lenses, and that reported the highest amount of protein, 3.7 mg/lens [21]. In the current study, the amount of extracted protein from control lenses was 9.7 mg/lens after three nights and 10.2 mg/lens after 13 nights of extended wear. The use of lenses on an extended wear basis in the current study could be the reason for higher amounts of total protein being extracted. SDS-PAGE revealed there was no difference in the type of protein deposited on Mel4-coated or control lenses after three or 13 nights of wear. It appeared that lysozyme was the major protein extracted from either of the lenses, which agrees with other studies using etafilcon A lenses [38,53,54].

The current study demonstrated that lipids could be extracted from both Mel4-coated lenses and control lenses, with the amount of lipid being 25–31 μg/lens after three nights of wear and decreasing slightly to 13–21 μg/lens after 13 nights of wear. Previous results with etafilcon A lenses found very similar concentrations of lipids that could be extracted from the lenses: 20 to 44 μg/lens [21,25]. The types of lipids that adsorbed were not examined, and it might be interesting to examine this in future studies. Overall, as Mel4-coated lenses tended to have lower amounts of proteins and lipids adsorbed to their surfaces during wear and there was no difference in the types of proteins that were absorbed to Mel4-coated or control lenses or between three nights and 13 nights of lens wear, it appears that deposition of proteins and lipids onto lenses did not reduce the effectiveness of the Mel4 coating. It would be interesting to determine whether there were differences in the denaturation of proteins in particular on Mel4 and uncoated lenses in future studies.

The current study also evaluated whether proteolytic cleavage of Mel4 on the lenses could have been the reason for the reduction in antimicrobial activity, even though a previous study had reported that incubation of melimine, the parent peptide of Mel4, with tears did not reduce its antimicrobial activity [55]. Several previous studies have noted that proteolytic cleavage is a major potential drawback in the application of antimicrobial peptides [56,57,58]. Tears contain a variety of proteases, including tryptase from mast cells and NSP4 from neutrophils that both have trypsin-like activity [30,34]. Trypsin cleaves amino acids from proteins at lysine and arginine residues when these are not linked to a C-terminal proline. In addition, tears and the artificial tear fluid used in a previous study produced a slight reduction in melimine activity [13]. Tears and the artificial tear fluid contain lactoferrin which has weak proteolytic activity [59] and can hydrolyse peptide bonds on the C-terminal side of arginine residues [60]. The amino acid sequence of Mel4 (KNKRKRRRRRRGGRRRR) would make it susceptible to digest by trypsin-like proteases, although there was the possibility that covalent attachment to the contact lens surface might make Mel4 resistant to such digestion. However, both free and surface-bound Mel4 were susceptible to trypsin digestion. This suggests that Mel4-coated contact lens surfaces were probably degraded during wear, with the degradation eliminating its antimicrobial activity after three nights of wear. Future studies are warranted to improve the protease resistance of Mel4. Such studies could include the use of D-amino acid substitutions at protease cleavage sites and/or by terminal amination and acetylation of amino acids [61]. Alternatively, Mel4 could be converted into a peptide mimic, as these have been shown to be resistant to proteases [62].

## 5. Conclusions

In conclusion, Mel4-coated contact lenses retain antimicrobial activity against *P. aeruginosa* and *S. aureus* for at least three nights of wear but lose the activity at six nights of wear. Mel4-coated lenses most likely lost activity during extended wear due to degradation of the Mel4 peptide and not due to the deposition of proteins or lipids onto the lenses. The fact that the lenses remained active for between three and six nights of wear indicates that they could be worn on a daily basis or six-night replacement schedule.

## Data Availability

Data from the study are available upon request.
